# Non-Human Primate Malaria Infections: A Review on the Epidemiology in Malaysia

**DOI:** 10.3390/ijerph19137888

**Published:** 2022-06-27

**Authors:** Nor Diyana Dian, Mohd Amirul Fitri A. Rahim, Sherwin Chan, Zulkarnain Md Idris

**Affiliations:** 1 Department of Parasitology and Medical Entomology, Faculty of Medicine, Universiti Kebangsaan Malaysia, Kuala Lumpur 56000, Malaysia; p103737@siswa.ukm.edu.my (N.D.D.); p103307@siswa.ukm.edu.my (M.A.F.A.R.); 2 Department of Microbiology, Tumor and Cell Biology, Karolinska Institutet, 17177 Stockholm, Sweden; sherwin.chan@ki.se

**Keywords:** malaria, *Plasmodium*, zoonotic, epidemiology, Malaysia

## Abstract

Malaria remains a public health problem in many parts of the world, including Malaysia. Although Malaysia has been recognized as one of the countries free from indigenous human malaria since 2018, the rising trend of zoonotic malaria, particularly *Plasmodium knowlesi* cases, poses a threat to public health and is of great concern to the country’s healthcare system. We reviewed previously scattered information on zoonotic malaria infections in both Peninsular Malaysia and Malaysian Borneo to determine the epidemiology and distribution of emerging zoonotic malaria infections. Given the high prevalence of zoonotic malaria in Malaysia, efforts should be made to detect zoonotic malaria in humans, mosquito vectors, and natural hosts to ensure the success of the National Malaria Elimination Strategic Plan.

## 1. Introduction

Malaria remains a public health problem in many parts of the world. It is a life-threatening disease caused by *Plasmodium* species parasites transmitted to humans through infectious bites of female *Anopheles* mosquitoes. It can be life-threatening if left untreated, especially among children under five years. In 2019, the World Health Organization (WHO) estimated 229 million malaria cases in 87 endemic countries, significantly reduced by 4% from 238 million cases in 2000 [1]. Despite the decline, approximately 627,000 malaria deaths were still recorded worldwide in 2020, with children under five and service disruptions during the COVID-19 pandemic accounting for 77% and 68% of all malaria deaths, respectively [2]. Malaria is still considered a public health problem in Malaysia and has been on the list of the national notifiable diseases since 1988 [3]. Since the early nineteenth century, it has been recognized as a serious disease in Peninsular Malaysia. It was a significant issue in the Straits Settlements (i.e., a division of British Malaya including Singapore, Penang, Malacca, Labuan, and some smaller islands). Malaria was responsible for one-third of all registered deaths in Penang in 1829 and 40,070 deaths in Peninsular Malaysia in 1944 [4]. The mortality rate due to malaria began to decline after World War II due to numerous antimalarial interventions. Malaria has also long been a problem in Malaysian Borneo (i.e., Sarawak and Sabah), with Sabah recording 250,000 human malaria cases in 1951 [4].

Malaria cases have decreased significantly in Malaysia since the Malaria Eradication Program in Malaysian Borneo and Peninsular Malaysia in 1961 and 1967, respectively [5]. It dramatically reduced malaria cases from 243,870 in 1961 to 4725 in 2012 [6]. Subsequently, the program was strengthened, resulting in zero indigenous human malaria cases (i.e., *Plasmodium vivax* and *Plasmodium falciparum*) since 2018 [1]. Despite the significant decrease in human malaria, the emergence of simian malaria is a major public health problem in the less developed areas of Malaysia. In particular, it is a problem among the hard-to-reach indigenous populations (i.e., Orang Asli) in Peninsular Malaysia and the remote interior communities in Malaysian Borneo [3,5,7,8]. In 2019, Malaysia recorded 3222 zoonotic malaria with six fatalities [9]. Furthermore, the influx of migrant workers from malaria-endemic countries and challenges of drug resistance have exacerbated the risk of re-emergence of the disease. Due to the large-scale clearing of forest areas for logging and agricultural purposes, Malaysia faces the problem of increasing cases of simian malaria driven by the migration of macaques to human settlements, particularly in the remote areas where the aboriginal populations live [3,5,8,10]. Although Malaysia has been recognized as one of the countries free from indigenous human malaria since 2018 [1], it is essential to acknowledge the prevalence of non-human malaria and strengthen the effectiveness of the national elimination program.

**Figure 1:**
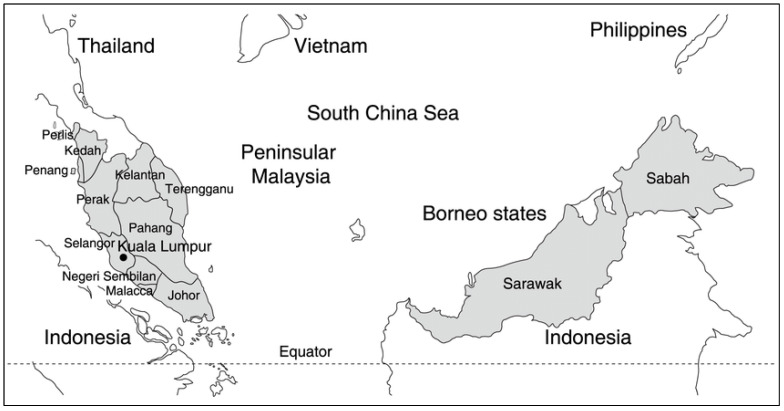
Map of Malaysia showing all states and federal territories.

Given the scattered information on non-human primate *Plasmodium* parasites in Malaysia, this review is intended to collate previous information on non-human malaria infections in different states of Malaysia (i.e., Peninsular Malaysia and Malaysian Borneo) (Figure 1), including its epidemiology and distribution.

## 2. Non-Human Primate Malaria Species

In Southeast Asia, at least 11 *Plasmodium* species infect non-human primates; five of these can be naturally found in macaques, while the remaining mainly infect apes [11]. Infection by these *Plasmodium* species usually results in very low parasitemia and causes mild or asymptomatic disease in their natural hosts. Since 1960, seven simian malaria species have been reported as transmissible to humans through mosquitoes, i.e., *Plasmodium cynomolgi*, *Plasmodium brasilianum*, *Plasmodium eylesi*, *Plasmodium knowlesi*, *Plasmodium inui*, *Plasmodium schwetzi*, and *Plasmodium simium* [12]. Three of these are known to pose a potential risk of zoonotic malaria in Southeast Asia. Other than *P. knowlesi*, which is now known to be the cause of the fifth human malaria [13], two other malaria species capable of infecting humans are *P. cynomolgi* and *P. inui* [14,15]. Both of them also share the same natural hosts with *P. knowlesi*, particularly the long-tailed (*Macaca fascicularis*) and pig-tailed (*Macaca nemestrina*) macaques [16].

Simian malaria parasites were first reported in Peninsular Malayan monkeys in 1908 [17]. Initially, it was assumed that simian malaria transmission to humans would not be possible. However, this assumption was dismissed when researchers in the Centers for Disease Control and Prevention laboratories Atlanta, USA, were accidentally infected with a simian malaria species via mosquito bites in the laboratory [15,17]. In 1965, the first natural human infection was reported in an American surveyor in Peninsular Malaysia [18].

## 3. *Plasmodium knowlesi*

In the 1930s, *P. knowlesi* was isolated and thoroughly studied for the first time. Napier and Campbell discovered it in the blood of a long-tailed macaque from Singapore in 1931 while investigating leishmaniasis [19]. They inoculated three macaques, two long-tailed macaques and a rhesus macaque (*Macaca mulatta*) with the infected blood, and subsequently, the rhesus monkey developed a severe infection [19]. In the following year, the blood form of the *P. knowlesi* parasite was described by Robert Knowles and his assistant, Das Gupta from the Calcutta School of Tropical Medicine in India. They performed serial-passage of infected blood from the infected monkeys from Napier and Campbell’s study. They also demonstrated the parasite’s ability to infect humans via blood inoculation [20]. In the same year, Colonel John Alexander Sinton, the then Director of the Malaria Survey of India, further investigated the parasite with his coworker Dr Mulligan. They identified specific morphological features of the blood-stage parasite and discovered its unique 24-h schizogonic cycle using the parasite isolated by Knowles and Das Gupta and the parasite they isolated from a long-tailed macaque in Singapore. These findings convinced them that it was a new *Plasmodium* species [21].

In 1935, Van Rooyen and Pile utilized *P. knowlesi* to treat patients with neurosyphilis. Patients who were previously infected with *P. vivax* were less susceptible than those who never had malaria [22]. In the following year, Chopra and Das Gupta successfully treated neurosyphilis in two patients by inoculating them with *P. knowlesi* from *M. fascicularis*, thus demonstrating the potential use of *P. knowlesi* in treating the disease [23]. Until the 1950s, malaria treatment on neurosyphilis patients had been highly effective in Romania. However, the practice was discontinued in 1955 after discovering that the parasite became more virulent after 170 blood transfers and required pharmacological treatment to terminate the infection [23].

The first evidence of *P. knowlesi* being naturally transmitted to humans was reported in 1965 [18,24]. After spending five days in a primary forest near Bukit Kertau, Pahang in Peninsular Malaysia, an American surveyor acquired the infection. On returning to the United States, he began to experience symptoms and was first diagnosed as being infected with *P. falciparum* by microscopy. He was then referred to the Army’s Walter Reed Hospital in Washington, D.C., and then to the National Institute of Health’s Clinical Centre in Bethesda, where he was diagnosed with *Plasmodium malariae* infection. His blood sample was given to a group of malariologists at the National Institutes of Health (NIH) investigating *P. malariae* and was used to inoculate volunteers at the US Penitentiary in Atlanta, Georgia, who subsequently developed malaria. Rhesus macaques were also inoculated with his blood, resulting in the death of all animals after developing severe illnesses.

Six years later, another human case of *P. knowlesi* infection was suspected based on presumptive diagnosis six years after the first report of natural human *P. knowlesi* infection [25]. The diagnosis was based on microscopy and serological tests. From then, no other case of *P. knowlesi* infection in humans was reported until a large cluster of infections in a community was first detected in the Kapit Division of Sarawak, Malaysian Borneo, in 2004 [24].

## 4. *Plasmodium cynomolgi*

Another simian malaria parasite, *P. cynomolgi*, is also deemed an emerging cause of malaria in humans. The zoonotic capability of *P. cynomolgi* has been proven through accidental and experimental infections [14,26,27]. Halberstadter and von Prowazek initially found this parasite in the blood samples collected from a cynomolgus monkey [28], commonly known as the crab-eating or long-tailed macaque (*M. fascicularis*) in Java in 1907. In the rhesus monkey, *P. cynomolgi* was found to behave similarly to *P. vivax*, with repeated relapses from an exoerythrocytic source, which later was found to be dormant “hypnozoites” in the liver. It, therefore, became the animal model for relapsing malaria. The potential of *P. cynomolgi* transmission from mosquito to human was initially demonstrated by the renowned entomologist Don Eyles, who became ill with malaria while studying mosquito transmission in laboratory primates [29,30].

It was previously thought that humans could not be naturally infected with primate malaria parasites. Since then, many artificial human infections have been studied [31,32,33]. *P. cynomolgi* is similar to *P. vivax* in morphology, genetics, and biology, albeit having a more extended incubation period of sporozoite-induced infections in humans [31]. *P. cynomolgi* do not cause severe malaria in experimentally infected individuals. Coatney et al. reported the persistence of initially symptomatic *P. cynomolgi* human infections for up to 58 days in untreated infections with both the M and B strains [31,32,33]. Like other malaria parasites, persistence in the blood can occur after symptomatic infection, or otherwise, newly acquired infections are almost always asymptomatic, especially if the host has prior exposure. This property can make humans a potential reservoir of the parasite.

*P. cynomolgi* has been used in several studies on malaria immunity, such as activation of the immune system components during the infection [34,35] and the parasite as a model for malaria-HIV co-infections [36]. The genome of *P. cynomolgi* encodes orthologues of the *vir*-gene family, which are responsible for immune evasion in *P. vivax* [37]. *P. cynomolgi* has been reported to induce strain-specific immunity, a characteristic seen in both human and rodent malaria parasites [38,39]. The species has also been studied to understand the interactions between *Plasmodium* and its vector. Furthermore, studies on mechanical characteristics of infections and their genetic basis in mosquitoes [40,41,42], and experiments towards understanding the factors regulating mosquito infectivity, have been conducted [43,44]. *P. cynomolgi* has also been used to test the efficacy of several new anti-malarial drugs [45,46,47,48,49] and prompted several studies on malaria evolution and genetic diversity [50,51,52].

*P. cynomolgi* is less restrictive than *P. knowlesi* in mosquito vector transmissibility [53]. Besides its natural vectors, *Anopheles cracens* and *Anopheles dirus* [54,55], it can also be transmitted by *Anopheles farauti* [56] and by species commonly raised in the laboratory, such as *Anopheles gambiae* and *Anopheles stephensi* [57].

## 5. *Plasmodium inui*

Besides *P. brasilianum*, *P. inui* is the only major non-human primate malaria parasite with a quartan life cycle [14]. While previously assumed to be closely related to *P. malariae*, recent phylogenetic analyses have included *P. inui* in the clade of primate malaria parasites that includes *P. vivax* [58]. Indeed, early immunological findings have suggested its distinction from the *P. malariae* subgroup [59]. *P. inui*, originally isolated from a Javan *M. fasicularis*, may infect a wide range of monkeys, including the New World Platyrrhini [60], and can be transmitted by a range of *Anopheles* species [61]. This simian malaria parasite has an extended period of development within the vector (i.e., 15 days), it also takes longer to develop during the liver stage (i.e., 9–10 days), as well as adopting a quartan (i.e., 72-h) period of development in the blood [14,60]. Furthermore, *P. inui* is prone to producing a long-term chronic infection in *M. mulatta* with blood-stage parasitemia lasting for 14 years or more [62,63]. Although parasitemia is low throughout chronic infections, kidney damage has been documented in animals reminiscent of nephrotic syndrome with chronic glomerulonephritis, which is similarly associated with *P. malariae* infection [64]. It is important to note that *P. inui*, especially the OS strain, can cause patent infections in humans [14,31], thus making it a possible zoonotic disease with medical significance [14].

## 6. *Plasmodium coatneyi*

*P. coatneyi* is a tertian malaria species found primarily in macaques in Southeast Asia and is closely related to *P. knowlesi* [65]. It is transmitted by Asian Anopheline mosquitoes such as *A. dirus* and *Anopheles freeborni*, while transmission by *A. stephensi* and *A. gambiae* have also been established, although less effective [66]. The evidence for the effective establishment of the erythrocytic cycle in New World monkeys is scarce; however, they appear to be susceptible to the liver stages of the parasite [67]. *P. coatneyi* liver stages have also been successfully cultured in vitro [68].

*P. coatneyi* shares some features with the malignant falciparum malaria in humans, i.e., presence of knob protrusions on the surface of infected erythrocytes, cytoadherence to the vascular endothelium, rosetting, and the induction of ‘cerebral malaria’ [69,70,71,72,73,74,75]. *P*. *coatneyi* has also been utilized in investigations involving co-infections with schistosomiasis [76] and provides an excellent model for studying the multisystemic dysfunction associated with severe malaria in monkeys [77].

## 7. Distribution of Knowlesi Malaria Species in Malaysia

In Malaysia, the first naturally acquired *P. knowlesi* in humans was reported in Pahang in 1965, followed by a second probable case, a few years later in Johor [25]. Both states are located in Peninsular Malaysia. Knowlesi malaria was believed to be a rare disease compared to other malaria species until a large cluster of human infections was discovered in Kapit, Sarawak, in 2004 [24]. Since then, the number of reported knowlesi infections has steadily risen in Sabah and Sarawak, primarily in the interior regions [78,79,80,81,82,83,84,85]. Based on Table 1, most cases of *P. knowlesi* malaria were detected in the states of Malaysian Borneo (i.e., Sabah and Sarawak).

The geographical topography of Sabah and Sarawak, which have vast forested areas, consist of primary and secondary forests that are habitats for the natural reservoir hosts (i.e., macaques) and the mosquito vector *Anopheles*. It was found that there is a significant positive correlation between forest density and the number of malaria cases [85]. The density of the forest is important as it increases contact between the human hosts and the habitats of *Anopheles* mosquitoes, thus increasing the chance for malaria transmission. Zoonotic malaria cases also increased due to the opening of new rubber estates, which necessitate the removal of previously dense forests to construct new roads and new laborers’ villages [85].

Table 1 shows studies conducted in Sabah and Sarawak to determine the prevalence and incidence of knowlesi malaria in the community using molecular PCR technique since it is more sensitive and specific than the standard method for malaria diagnosis [109,110,111,112,113]. PCR proved superior to microscopic examination in detecting human malaria parasites because infected erythrocytes were only commonly observed from blood films with parasitemia above 100,000 parasites/L blood [88,114]. Thus, submicroscopic infections would often give false-negative results. In addition, *P. knowlesi* parasite shares similar morphological characteristics with *P. falciparum* in the early trophozoite stage and *P. malariae* in the later stages of the erythrocytic cycle, which may lead to the misdiagnosis of the causative species [114,115]. A substantial number of *P. knowlesi* infections have been documented in Sarawak, Malaysian Borneo [24,87,94,98,99,101,102,103,105,114], and in other Southeast Asian countries such as Myanmar [116], Thailand [117,118,119,120,121], the Philippines [122,123,124], and Singapore [125,126,127]. Therefore, *P. knowlesi* infection in humans is not as uncommon as previously thought, its prevalence is high among the community, and most cases are asymptomatic. Several epidemiological studies conducted in Sarawak had found that *P. knowlesi* parasite is the main contributor to the total malaria cases (Table 1). It is in line with previously published data that found a high incidence of *P. knowlesi* malaria in Sarawak [93]. These studies revealed a higher tendency for *P. knowlesi* than other *Plasmodium* species among populations in Sarawak. Raja et al. detected 815 (77.8%) *P. knowlesi* mono-infection cases out of 1047 positive malaria patients from Kapit Hospital, Sarawak, using a molecular method [103], which gives higher sensitivity than the conventional microscopic examination. In addition, several submicroscopic *P. knowlesi* infections were detected in asymptomatic individuals, as reported by Jiram et al. and Siner et al. [98,101] (Table 1). This is consistent with a study on asymptomatic and/or low-density malaria infection in Malaysia [128]. Accurate diagnosis of asymptomatic submicroscopic malaria is critical in reflecting the actual malaria burden and avoiding ineffective interventions because low levels of parasitemia may serve as a hidden transmission reservoir, thus remaining infectious to susceptible mosquito vectors. The rising incidence of *P. knowlesi* in Sarawak and the lack of indigenous human malaria cases since 2018 [1] depicts the malaria trend in Malaysian Borneo and Peninsular Malaysia. Furthermore, the highest number of *P. knowlesi* in Malaysia was contributed mainly from *P. knowlesi* cases notified in Sabah and Sarawak [105]. The knowlesi malaria occurrence is the highest in Sarawak, possibly due to deforestation and logging activities in some interior regions. Since the forest is a natural habitat for macaques, deforestation displaces macaques to areas near human settlements and increases the risk of parasites transmission from macaques to humans.

In Malaysia, knowlesi malaria cases are not limited to Sabah and Sarawak in the Malaysian Borneo. Cases are also reported in states with dense forest coverage in Peninsular Malaysia, such as Kelantan, Perak, and Pahang [129]. Although epidemiological reports from Peninsular Malaysia were fewer than in Malaysian Borneo, studies showed that *P. knowlesi* is present in most states in the peninsular (Table 1). As reported by Noordin et al., who conducted a study in four states with the highest incidence rate of malaria in Peninsular Malaysia, 10 out of 19 positive malaria cases were caused by *P. knowlesi*. In addition, Braima et al. and Vythilingam et al. detected 75 (4.6%) and 65 (58.6%) knowlesi malaria among the communities in Peninsular Malaysia, respectively (Table 1). *P. knowlesi* and *P. malariae* can be distinguished with better molecular diagnostic methods. The utilization of molecular diagnostics would better elucidate the extent and coexistence of *P. knowlesi* in humans, nonhuman primates and vectors.

## 8. Distribution of *cynomolgi*, *inui* and *coatneyi* Malaria Species in Malaysia

Although 92% of all malaria cases in Malaysia have been due to knowlesi malaria [2], there have also been some cases involving other species of simian malaria. Since 2014, the cases involving simian malaria other than *P. knowlesi* have been reported to occur naturally in humans. Table 2 shows that *P. cynomolgi* infection had been recorded in humans in Malaysian Borneo and Peninsular Malaysia. The first case of natural human cynomolgi malaria infection was recorded on the east coast of Peninsular Malaysia (i.e., Hulu Terengganu) in 2014 [12]. Initially, the case was microscopically diagnosed as *P. vivax* infection but was later confirmed by molecular PCR method as *P. cynomolgi*. Yap et al. and Grignard et al. reported nine and two mono-infection cases of *P. cynomolgi* in their studies, respectively [100,108]. As seen in Table 2, *P. cynomolgi* infection was more commonly reported in humans than *P. coatneyi* and *P. inui*. PCR detected these cases among asymptomatic individuals in the communities. It correlates with most studies on human *P. cynomolgi* infection, which unanimously observed only mild clinical symptoms in infected individuals, if not asymptomatic [29,32,100,103,130]. Due to the limited disease severity and its morphological similarity to *P. vivax*, accurate microscopic speciation is challenging to make, and the actual incidence rate of *P. cynomolgi* is likely to be significantly higher than what is currently thought. Indeed, many patients in these studies with *P. cynomolgi* mono-infection would have been misdiagnosed as *P. vivax* infections if they had relied only on microscopy or rapid diagnostic test [12,103,130,131,132]. Thus, human infections caused by *P. cynomolgi* are potentially widely distributed in Malaysia.

*P. inui* was experimentally reported to infect humans in 1938, with a subsequent report in 1966 [14]. Liew et al. reported two out of 71 cases of asymptomatic *P. inui* mono-infections in Peninsular Malaysia (Table 2) using nested PCR on concentrated high-volume blood samples. Previously, natural human *P. inui* infection had not been described [31], even though the parasite is experimentally transmissible to humans [98]. Inui malaria would have been misdiagnosed by microscopy as human malaria parasite since it shares morphological similarities with *P. malariae* [31,134].

Table 2 shows three coatneyi malaria infections out of 102 *Plasmodium* genus positive cases reported by Yap et al. [108]. It proved that *P. coatneyi* infects monkeys and causes natural infection in humans, thus making it a zoonotic disease even though previous attempts at infecting humans using the blood of rhesus monkeys infected with *P. coatneyi* parasite or via infected mosquitoes were unsuccessful.

The risk of acquiring simian malaria infection is high among the communities living near forest fringes or those who work inside the forest due to the proximity to reservoir hosts and mosquito vectors. High-risk groups include indigenous people, farmers, cultivators, and forest workers [135,136]. Human infection by zoonotic malaria occurs in areas where the vectors and the natural host species co-exist [137]. Local ecological changes, such as deforestation and biodiversity loss, are possible drivers for increased interconnectivity between humans, the reservoir host, and the vector, altering the transmission dynamics of zoonotic malaria infection [97,138]. Understanding the impact and mechanisms of biodiversity loss in an ecosystem on the risk of infectious disease acquisition is a multi-factorial problem [139].

The rapid development of industrial plantations is responsible for mass deforestation in Malaysia, and this trend is expected to continue [140]. It will potentially cause ecosystem imbalance and increase the threat of zoonotic disease outbreaks [141]. The loss of habitat diversity, increasing forest fragmentation, and forest decimation due to deforestation may influence the prevalence of simian malaria parasites and alter the behavior and transmission dynamics between macaques, vectors, and humans [141,142,143].

The social and economic factors that enabled the rapid conversion of forest areas to industrial lands have also been identified [144]. By establishing forest fragments and fringes, the modification of local forested landscapes for anthropogenic use has been proven to provide ideal settings for mosquito populations harboring simian malaria parasites [145,146]. Deforestation impacts the population distribution and density of the macaque host reservoir [97,138], resulting in their invasion into human settlements [142]. Similar to the situation with *P. knowlesi*, the epidemiology of the *P. cynomolgi*, *P. inui,* and *P. coetneyi* malaria species are significantly affected by forest-clearing activities.

## 9. Population at Risk

Over the past few years, researchers have been looking at the relationships between environmental, occupational, sociodemographic, and domestic factors that may lead to an increased risk of zoonotic malaria infection, particularly with *P. knowlesi*. Understanding and identifying these risk variables will aid in the development of appropriate and effective public health interventions for knowlesi malaria. The use of molecular diagnostics has increased the capacity to identify *P. knowlesi* malaria, and current studies show ongoing environmental and ecological changes affecting its epidemiology. Among the main causes of the increasing number of *P. knowlesi* malaria cases in Malaysian Borneo include deforestation, reduced biodiversity, and migration of macaques to forest fringes; these developments have increased contact among humans, mosquito vectors, and macaques [84,97,138,143,147,148]. Fornace et al. found that factors such as more than 65% forest coverage within a 2 km radius, more extensive historical forest loss, and lower elevation are associated with increased *P. knowlesi* infection [97]. They hypothesized that the association could be related to changes in human habitat and changes in the macaque and mosquito environment due to deforestation and agricultural activities. Furthermore, previous studies have also found that farmers, oil palm plantation workers, and people clearing agricultural areas are at high risk of contracting *P. knowlesi* infection [82,83]. Those living in the habitat of macaques and anopheline vectors of *P. knowlesi* are also at high risk of infection. Most knowlesi malaria patients in Malaysian Borneo, where most cases have been reported, are adults who work as farmers, hunters, or logging camp workers [24,86]. In Vietnam, it was reported that those at risk live in the forest fringes, collect bamboo and rattan, and work on their farms on the hillsides [149]. Thus, the present high incidence of *P. knowlesi* infection in Malaysian Borneo can be attributed to its geographical area and the presence of the primary mosquito vectors [150].

## 10. Mosquito Vectors

The vectors of knowlesi malaria are forest-dwelling mosquitoes that belong to the *Anopheles* Leucosphyrus group; hence, mosquito population dynamics are an important predictor of malaria risk. As mentioned above, deforestation is one of the significant factors contributing to the increase in the incidence of *P. knowlesi* infection in Malaysia [135]. However, successful simian malaria transmission can only occur if humans, natural reservoir hosts (i.e., macaques), and competent mosquito vectors are brought into contact. The initial stage in developing control strategies is to identify the vector species responsible for simian malaria transmission and the environments linked with their exposure to humans. The vectors of *P. knowlesi* malaria in Malaysia comprise five *Anopheles* species of the Leucosphyrus group, namely, *Anopheles hacker*, *Anopheles latens*, *Anopheles cracens*, *Anopheles introlatus*, and *Anopheles balabacensis* [151,152,153,154,155]. These vectors are found mainly in the forests and are outdoor biters. *A. balabacensis* has been confirmed as the principal vector in the biggest hotspot of human infection in the Kudat district of Sabah [156]. *A. latens* has been identified as the vector in Kapit, Sarawak, where most human knowlesi malaria cases have been reported. This species prefers to feed on macaques at a higher location and feeds in the forest primarily between 7 and 10 p.m. [152]. It is attracted to both long-tailed macaques and humans [152]. Moreover, a study by Ang et al. found the presence of *A. balabacensis* and *A. donaldi* in Lawas, northern Sarawak [157]. Meanwhile, *A. cracens* is the main malaria vector of the knowlesi parasite in most states in Peninsular Malaysia, with a peak biting time between 8 and 9 p.m. [55]. This species is highly zoophilic and has been found to feed on macaques at the canopy level and humans at the ground level. Land use and land cover change (LULCC) directly impact anopheline mosquito populations, altering the abundance, species composition, and life cycle. Ecological changes in soil, sunshine coverage, types of plantations, water pocket development, and water temperature affect the breeding behaviors of *Anopheles* malaria vector, with the effects varying among *Anopheles* species [158]. Deforestation reduces shaded water bodies, the preferred breeding ground for some *Anopheles* species. Other *Anopheles* species thrive in water bodies with more sunlight which can increase larval survival, adult productivity, and intrinsic growth rates and shorten the gonotrophic cycles to increase the vectorial capacity [159]. Furthermore, environmental and climatic changes caused by LULCC may favor the survival of several *Anopheles* species, permitting long-term malaria transmission or affecting the availability of hosts and blood meals [158]. Temperature rises and changes in rainfall patterns may result in a more extended malaria season in many sub-Saharan African regions and affect the local vectorial capacity [160]. Thus, climate changes affect the transmission dynamics of simian malaria, influencing the parasite density in humans, mosquito vectors, and natural reservoir hosts.

## 11. Reservoir Hosts

Humans can acquire knowlesi malaria when the habitats of humans, macaques, and competent mosquito vectors overlap, mainly due to various human activities that destroy the natural forest ecosystem. Subsequently it can lead to an increase in the incidence of zoonotic malaria in Malaysia. Long-tailed (*M. fascicularis*) and pig-tailed (*M. nemestrina*) macaques from Singapore [20] and Peninsular Malaysia [55] have been identified as reservoir hosts of *P. knowlesi*. Macaque populations tend to wander around village areas (average distance of 6 km), and areas inside the forest with lower elevation and warmer temperatures [137]. A single *P. knowlesi* infection from a leaf monkey (*Presbytis melalophos*) from Peninsular Malaysia [161] has also been reported. The presence of monkeys in peri-domestic areas is a substantial predictor of risk, indicating that monkey to human transmission is still the most common mode of transmission, rather than human to human transmission.

## 12. Discussion

Although Malaysia has successfully eliminated indigenous human malaria species [1,3,7,8], *P. knowlesi* malaria continues to infect many people in the country’s rural areas [84,92,99]. In Sarawak (Malaysian Borneo), zoonotic malaria caused by *P. knowlesi* infections was studied extensively from 2004 [24] and was eventually found to be a common cause of malaria [162,163]. Besides *P. knowlesi*, increasing evidence of naturally acquired cynomolgi infection in humans was also documented in Malaysia [132], highlighting the risk of zoonotic malaria infections in the country.

Successful transmission can only occur if the competent vectors and the natural hosts co-exist in proximity, leading to a rising number of zoonotic malaria cases. Humans and macaques can share the same habitat, particularly those who practice agriculture or farming near the forests or visitors who encounter macaques in their natural habitat. The availability of appropriate vectors and the close contact and geographical distribution between humans and macaques can lead to malarial disease transmission between the two populations [137,164,165]. Zoonotic malaria infection in humans is influenced by many factors, including anthropogenic LULCC that inadvertently alters the transmission dynamics among the macaque reservoirs, the vectors, and the people, as seen in Malaysian Borneo [158,166]. Macaques, who have lost their natural habitats due to deforestation for palm oil plantations or other clearing activities, are then coerced to encroach on human settlements resulting in a rise in the prevalence of zoonotic malaria infection in humans [83].

It is vital to address zoonotic malaria as one of the threats to public health problems in Malaysia. Zoonotic malaria cases are widely reported in Malaysian Borneo, as concluded by many studies [7]. In Peninsular Malaysia, only 13 epidemiological studies have been performed over the past 40 years, most of which have still relied on conventional microscopy [7]. This could lead to an underrepresentation of the actual incidence of zoonotic malaria in the community. Further studies and continued surveillance of zoonotic malaria parasites in Malaysia are necessary, using sensitive molecular methods to obtain accurate and reliable data for monitoring these emerging zoonotic infections.

## 13. Conclusions

In conclusion, zoonotic malaria constitutes a public health concern in Malaysia and other Southeast Asian countries. The natural habitats of the hosts and vectors and the evolution of the parasite could drive the transmission of this neglected but emerging disease. Moreover, the global environmental and climate changes could affect the dynamics of transmission of simian malaria as an emerging human cause of malaria. Further studies using molecular and multi-disciplinary approaches to detect simian malaria infection in humans, vectors, and natural hosts are necessary to ensure the success of the National Malaria Elimination Strategic Plan in Malaysia.

## Figures and Tables

**Table 1:** Distribution of *P. knowlesi* parasite reported in Malaysia.

Publication Year	Study Area (States) in Malaysia	Sampling Year	Study Design	No. of Blood Samples Tested	No. Positive *Plasmodium* spp.	Incidence or Prevalence of *P. knowlesi* (%)	References
2004	Sarawak	2000–2002	Prospective	208	208	106 (51)	[24]
2008	Perlis, Kedah, Pulau Pinang, Perak, Kelantan, Terengganu, Pahang, Selangor, Melaka, Negeri Sembilan, Johor, Kuala Lumpur	2005–2008	Prospective	111	111	65 (59)	[55]
2008	Sabah, Sarawak, Pahang	2001–2006	Prospective	1014	1014	280 (28)	[86]
2009	Sarawak	2006–2008	Prospective	169	169	107 (63)	[87]
2009	Sarawak	1996	Retrospective	47	36	29 (62)	[88]
2011	Sabah	2010	Retrospective	243	107	63 (26)	[78]
2011	Sabah	2007–2009	Prospective	78	78	56 (72)	[89]
2012	Sabah	2009–2011	Retrospective	18,993	445	339 (2)	[85]
2013	Selangor	2006–2012	Prospective	1623	1623	75 (5)	[90] *
2013	Sabah	2008–2011	Prospective	189	189	42 (22)	[91]
2013	Sabah	1992–2011	Prospective	14618	14,618	2181 (15)	[92]
2013	Sabah	2010–2011	Retrospective	387	295	130 (34)	[79]
2014	Sabah	2010–2013	Retrospective	1366	1082	924 (68)	[80]
2014	Sabah, Sarawak, Perlis, Kedah, Pulau Pinang, Perak, Kelantan, Terengganu, Pahang, Selangor, Melaka, Negeri Sembilan, Johor, Kuala Lumpur	2012–2013	Retrospective	457	453	256 (56)	[93]
2014	Sarawak	2010–2011	Prospective	40	40	28 (70)	[94]
2015	Sabah	2012–2013	Prospective	207	207	152 (73)	[95]
2016	Sabah	2012–2013	Retrospective	129	109	67 (52)	[96]
2016	Sabah	2012–2014	Retrospective	1147	206	20 (2)	[97]
2017	Sarawak	2014–2015	Cross-sectional	3002	8	7 (0.3)	[98]
2017	Sarawak	1992–2014	Prospective	9364	9364	9364 (100)	[99]
2019	Sabah	2015	Cross-sectional	876	54	3 (0.3)	[100]
2019	Sarawak	2013–2014	Cross-sectional	251	118	9 (4)	[101]
2020	Sabah, Sarawak, Perlis, Kedah, Pulau Pinang, Perak, Kelantan, Terengganu, Pahang, Selangor, Melaka, Negeri Sembilan, Johor, Kuala Lumpur	2000–2018	Prospective	117,224	117,224	19,975 (17)	[102]
2020	Sarawak	2013–2017	Prospective	1047	1047	815 (78)	[103]
2020	Johor, Pahang, Kelantan, Selangor	2019–2020	Cross-sectional	585	19	10 (2)	[104]
2020	Sabah, Sarawak, Perlis, Kedah, Pulau Pinang, Perak, Kelantan, Terengganu, Pahang, Selangor, Melaka, Negeri Sembilan, Johor, Kuala Lumpur	2013–2017	Prospective	16,500	16,500	11,380 (69)	[105]
2020	Sabah	2015–2017	Prospective	3867	3867	3524 (91)	[84]
2020	Sabah	2016	Cross-sectional	1222	410	340 (28)	[106] *
2021	Sabah, Sarawak, Kuala Lumpur, Perak, Pahang, Pulau Pinang, Terengganu	2015–2016	Retrospective	112	80	54 (48)	[107]
2021	Sarawak, Pahang, Perak, Selangor, Negeri Sembilan, Melaka, Kelantan	2011–2014	Retrospective	645	102	40 (6)	[108]

* Cases detected only by microscopy examination with no PCR performed.

**Table 2:** Published studies of *P. cynomolgi*, *P. inui*, and *P. coatneyi* in Malaysia.

Publication Year	Study Area (i.e., State in Malaysia)	Sampling Year	Study Design	No. of Blood Samples Tested	No. of Positive *Plasmodium* spp.	No. of Simian Malaria Species, (n)	References
2014	Terengganu	2011	Case report	1	1	*P. cynomolgi*	[12]
2019	Terengganu	2018	Case report	1	1	*P. cynomolgi*	[131]
2019	Sabah	2015	Cross-sectional	876	54	*P. cynomolgi* (2)	[100]
2020	Sarawak	2013–2017	Prospective	1047	1047	*P. cynomolgi* (6)	[103]
2021	Pahang, Perak, Selangor, Negeri Sembilan, Melaka, Kelantan, Sarawak	2011–2014	Retrospective	645	102	*P. cynomolgi* (9), *P. coatneyi* (3), and *P. inui* (3)	[108]
2021	Pahang	2020	Cross-sectional	71	2	*P. inui* (2)	[133]

## Data Availability

Not applicable.
